# Comparative transcriptomic analysis of maize ear heterosis during the inflorescence meristem differentiation stage

**DOI:** 10.1186/s12870-022-03695-6

**Published:** 2022-07-18

**Authors:** Xia Shi, Weihua Li, Zhanyong Guo, Mingbo Wu, Xiangge Zhang, Liang Yuan, Xiaoqian Qiu, Ye Xing, Xiaojing Sun, Huiling Xie, Jihua Tang

**Affiliations:** 1 National Key Laboratory of Wheat and Maize Crop Science, College of Agronomy, Henan Agricultural University, Zhengzhou, 450002 China; 2 Henan Institute of Crop Molecular Breeding, Henan Academy of Agricultural Sciences, Zhengzhou, 450002 China; 3 The Shennong Laboratory, Zhengzhou, Henan 450002 China

**Keywords:** Maize (*Zea mays* L.), Heterosis, Inflorescence meristem, Transcriptomics, Additive and non-additive gene expression, Allele-specific expression

## Abstract

**Background:**

Heterosis is widely used in many crops and is important for global food safety, and maize is one of the most successful crops to take advantage of heterosis. Gene expression patterns control the development of the maize ear, but the mechanisms by which heterosis affects transcriptional-level control are not fully understood.

**Results:**

In this study, we sampled ear inflorescence meristems (IMs) from the single-segment substitution maize (*Zea mays*) line lx9801^*hlEW2b*^, which contains the heterotic locus *hlEW2b* associated with ear width, as well as the receptor parent lx9801, the test parent Zheng58, and their corresponding hybrids Zheng58 × lx9801^*hlEW2b*^ (HY) and Zheng58 × lx9801 (CK). After RNA sequencing and transcriptomic analysis, 2531 unique differentially expressed genes (DEGs) were identified between the two hybrids (HY vs. CK). Our results showed that approximately 64% and 48% of DEGs exhibited additive expression in HY and CK, whereas the other genes displayed a non-additive expression pattern. The DEGs were significantly enriched in GO functional categories of multiple metabolic processes, plant organ morphogenesis, and hormone regulation. These essential processes are potentially associated with heterosis performance during the maize ear developmental stage. In particular, 125 and 100 DEGs from hybrids with allele-specific expression (ASE) were specifically identified in HY and CK, respectively. Comparison between the two hybrids suggested that ASE genes were involved in different development-related processes that may lead to the hybrid vigor phenotype during maize ear development. In addition, several critical genes involved in auxin metabolism and IM development were differentially expressed between the hybrids and showed various expression patterns (additive, non-additive, and ASE). Changes in the expression levels of these genes may lead to differences in auxin homeostasis in the IM, affecting the transcription of core genes such as *WUS* that control IM development.

**Conclusions:**

Our research suggests that additive, non-additive, and allele-specific expression patterns may fine-tune the expression of crucial DEGs that modulate carbohydrate and protein metabolic processes, nitrogen assimilation, and auxin metabolism to optimal levels, and these transcriptional changes may play important roles in maize ear heterosis. The results provide new information that increases our understanding of the relationship between transcriptional variation and heterosis during maize ear development, which may be helpful for clarifying the genetic and molecular mechanisms of heterosis.

**Supplementary Information:**

The online version contains supplementary material available at 10.1186/s12870-022-03695-6.

## Background

Heterosis is the phenomenon in which hybrid offspring are more vigorous than either parent, resulting in superior growth potential, yield capacity, adaptability, and stress resistance [1]. Heterosis is an important method for improving crop yield and quality, and it plays a critical role in the breeding of several crops, including maize, rice, sorghum, and rape [2–5]. However, the molecular mechanisms of heterosis remain unclear. At present, three classical hypotheses, dominance, overdominance, and epistasis, have been proposed to explain heterosis, and they have been debated for over 100 years [6]. With advances in science and technology, studies on heterosis using genomics, transcriptomics, proteomics, and epigenetics approaches have provided new insights into the molecular mechanisms of heterosis.

At the transcriptional level, changes in gene expression cause changes in biological regulatory networks, which are important sources of phenotypic novelty and affect heterosis [7]. When comparing differences in gene expression between parents and hybrids, multiple modes of gene action, including additivity, non-additivity, high- and low-parent dominance, and over- and underdominance, have been proposed to contribute to the phenomenon of heterosis [8]. Several studies in maize (*Zea mays*) revealed that additive effects are universal and are positively correlated with yield heterosis. Dominant and over-dominant expression patterns, which belong to the non-additive category, are also considered to be important factors in hybrid heterosis [9–11]. These changes in gene expression may alter biological regulatory networks, thereby affecting heterosis. By comparing the gene expression levels of hybrids and their parents at the maize ear developmental stage, Huang et al. [12] found that most negatively dominant genes are involved in carbohydrate metabolism, lipid metabolism, energy metabolism, and protein degradation, whereas positively dominant genes are mainly involved in DNA replication and repair. In allotetraploid *Arabidopsis thaliana*, non-additively expressed genes are significantly enriched in processes related to energy, metabolism, stress response, and plant hormone signal transduction [13].

In diploid hybrids, each gene is present in two copies, one each from the male and female parent. In theory, the alleles from both parents should be expressed equally in the hybrid. However, the transcriptional activities of different alleles in hybrids vary greatly [14, 15]. Allele-specific expression (ASE) refers to the preferential expression of a specific parental allele in the hybrid, driven by regulatory factors from the parental genomes [16]. Hybridization produces an extremely large pool of allelic variants, which affect gene expression levels. The expression differences caused by ASE may lead to phenotypic diversity, depending on the gene functions [17]. The ASE phenomenon has been documented in *Arabidopsis*, rice, maize, and barley [18–20]. ASE patterns may have distinct implications for the genetic basis of heterosis, especially for the dominance and overdominance hypotheses, because genetic variations frequently cause differential gene expression, which may lead to phenotypic differences in the hybrids [17, 21–23]. Although many genes in numerous species have been shown to exhibit ASE at the whole-genome level, the potential relationship between ASE and heterosis remains unclear.

Important traits related to maize yield, such as kernel row number, kernel number per ear, ear width, and ear length, are all determined during inflorescence meristem (IM) development. The development of immature maize ears displays strong heterosis in ear architectural traits, which greatly affect grain yield [24]. The size of the IM is significantly positively correlated with ear width and length, and its development directly affects the final morphological characteristics of the mature maize ear [25]. The classic pathway for maintenance of the IM amplification process is the *CLAVATA–WUSCHEL* (*CLV–WUS*) negative feedback loop. This pathway affects IM development by regulating the relationship between stem cell proliferation and tissue and organ differentiation [26]. *WUS* is a crucial regulator that determines stem cell formation and maintenance [27], and *CLV3* is a peptide ligand for the *CLV1/CLV2* receptor complex. Its expression may be induced by *WUS*, and it can move back to the organizing center to inhibit *WUS* expression. The *CLV3/WUS* negative feedback loop may affect the IM differentiation process [28]. The *WUS–CLV* feedback loop has also been tightly connected to auxin signaling, and auxin accumulates at specific positions to induce organ emergence in the peripheral zone [29]. Thus, hormones may play critical roles in the regulation of immature maize ear development.

To eliminate the influence of genetic background and reduce environmental effects, single-segment substitution line populations have been developed and used to map heterotic loci. Yu et al. [30] used single-segment substitution line test populations to research the heterosis performance of yield-related traits, and they discussed the advantages of single-segment substitution lines for heterosis research. Wang et al. [31] identified 21 yield-related heterotic loci by comparing differences between a test cross population and the background parents of 66 rice single-segment substitution lines. Wei et al. [32] used the same experimental design to identify 21 heterotic loci related to maize plant architectural traits.

During the development of the maize ear inflorescence, the IM stage is critical for ear development and heterosis. In the present study, we collected immature maize ears from the single-segment substitution line lx9801^*hlEW2b*^ (which contains the heterotic locus *hlEW2b* associated with ear width), the receptor parent lx9801, the test parent Zheng58, and their corresponding hybrids Zheng58 × lx9801^*hlEW2b*^ and Zheng58 × lx9801 during the IM stage. We then constructed global gene expression profiles using RNA sequencing technology (RNA-seq) to investigate the mechanisms of heterosis associated with transcriptome changes. Our research provides new insights into the relationship between transcriptomic changes and heterosis during maize ear development.

## Results

### Phenotypic analysis of near-isogenic lines and their corresponding hybrids

**Fig. 1:**
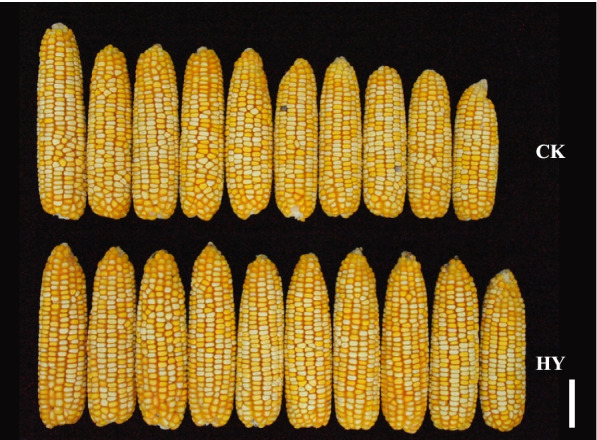
Heterosis performance of mature ear width trait between Zheng58×lx9801^*hlEW2b*^ (HY) and Zheng58×lx9801 (CK). Scale bar is 5 cm

We previously identified the chromosome segment substitution line sub-CSSL_16_ that carries the ear-width heterotic locus *hlEW2b* and contains a 1.98-Mb donor segment from Chang7-2. Ear width and weight are significantly higher in Zheng58 × sub-CSSL_16_ than in the control hybrid Zheng58 × lx9801 (*P* < 0.01) [33]. A heterotic effects analysis revealed that the heterotic locus *hlEW2b* exhibits strong over-dominance for heterosis (d/a ≥ 1, d = 4.19, a = 0.07) [33]. In the present study, the chromosome segment substitution line sub-CSSL_16_ containing the ear-width heterotic locus is redefined as lx9801^*hlEW2b*^. The phenotypic values of ear-related traits in the near-isogenic lines lx9801 and lx9801^*hlEW2b*^ and their corresponding hybrids Zheng58 × lx9801 (CK) and Zheng58 × lx9801^*hlEW2b*^ (HY) were investigated in multiple environments to evaluate the effect of the introgressed locus. In six environments, ear width and ear weight were significantly higher in HY than in CK, with average increases of 2.1 mm and 15.96 g, respectively, and their average over-standard heterotic values were 5.55% and 8.03% (Fig. 1; Table 1). There were no significant differences in five ear traits between the inbred lines lx9801 and lx9801^*hlEW2b*^ in two environments (Supplementary Table S1). These results imply that there may be a heterotic locus associated with the Zheng58 allele in lx9801^*hlEW2b*^ that controls the heterotic performance of ear width and weight in the hybrid.

**Table 1:** Comparison of ear traits between Zheng58 × lx9801 and Zheng58 × lx9801^*hlEW2b*^ in six environments

Traits	Env.	Zheng58×lx9801	Zheng58×lx9801^***hlEW2b***^	P-value	Over-standard heterosis
**Ear width (mm)**	E1	50.71±1.00	52.70±1.30**	1.17E-03	3.92%
	E2	46.16±0.96	47.64±1.32*	1.04E-02	3.21%
	E3	51.24±0.65	53.08±1.11***	2.65E-04	3.59%
	E4	50.09±1.43	53.12±1.26***	8.62E-05	6.00%
	E5	51.18±1.50	55.49±1.23***	6.43E-07	8.43%
	E6	52.22±1.61	56.47±1.45***	5.21E-07	8.14%
	Average	50.27±1.19	53.08±1.28**	1.99E-03	5.55%
**Axile width (mm)**	E1	29.07±0.37	31.67±0.24*	2.90E-02	8.94%
	E2	33.80±1.23	34.33±0.97*	4.24E-02	1.57%
	E3	33.49±1.41	35.36±2.09*	1.70E-02	5.58%
	E4	33.46±1.58	34.52±1.08*	4.04E-02	3.17%
	E5	31.56±1.03	32.33±0.94	3.90E-01	2.44%
	E6	32.93±1.16	35.44±1.25**	4.39E-03	7.62%
	Average	32.39±1.13	33.94±1.10	8.72E-02	4.89%
**Ear length (cm)**	E1	19.18±0.77	19.25±0.78	8.43E-01	0.30%
	E2	18.56±1.14	19.10±0.75	2.27E-01	2.90%
	E3	19.44±0.85	18.86±1.30	9.14E-02	-2.98%
	E4	18.35±1.49	17.58±0.93	1.83E-01	-4.19%
	E5	17.24±1.20	17.90±0.96	7.50E-02	3.80%
	E6	16.99±1.24	17.27±0.73	6.38E-01	1.60%
	Average	18.29±1.12	18.33±0.91	3.43E-01	0.24%
**Kernel row number**	E1	13.80±1.48	14.40±0.84	2.79E-01	4.35%
	E2	13.60±1.26	13.40±1.65	7.64E-01	-1.46%
	E3	13.40±1.65	13.20±1.40	7.73E-01	-1.40%
	E4	12.80±1.03	13.20±1.03	3.98E-01	3.13%
	E5	13.11±1.45	13.79±1.03	2.76E-01	5.20%
	E6	13.71±1.36	14.51±1.33	4.54E-01	5.85%
	Average	13.40±1.37	13.75±1.21	4.91E-01	2.61%
**Kernel number per row**	E1	36.00±3.02	34.60±1.65	2.14E-01	-3.80%
	E2	38.70±1.16	37.20±2.35	8.68E-02	-3.80%
	E3	38.70±1.89	37.30±2.36	1.60E-01	-3.61%
	E4	37.20±1.99	38.40±2.01	1.96E-01	3.23%
	E5	36.69±2.91	37.76±2.08	9.91E-02	2.90%
	E6	36.46±2.58	35.20±2.90	7.76E-02	-2.60%
	Average	37.29±2.26	36.74±2.23	1.39E-01	-1.28%
**Ear weight (g)**	E1	181.00±22.35	204.00±10.75**	8.87E-03	12.70%
	E2	118.93±5.79	125.45±6.38*	2.78E-02	5.49%
	E3	211.00±13.70	225.00±14.34*	3.85E-02	6.64%
	E4	195.00±13.54	216.00±24.13*	2.74E-02	10.77%
	E5	169.67±10.29	189.62±14.32**	6.07E-03	10.20%
	E6	201.97±11.62	212.35±8.80*	1.70E-02	5.14%
	Average	179.44±12.88	195.40±13.12*	2.09E-02	8.03%

Note: E1, E2, E3, E4, E5 and E6 are Xinxiang 2015, Hebi 2015, Xinxiang 2016, Hebi 2016, Xinxiang 2018 and Hebi 2018 China, respectively. *, ** and *** indicate significant differences at the 0.05, 0.01 and 0.001 probability level

**Fig. 2:**
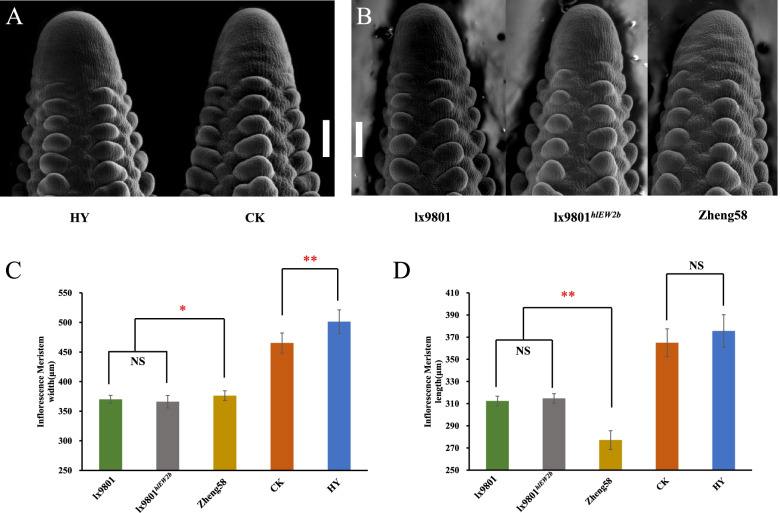
Inflorescence meristem size in hybrids and inbred lines. **A** Scanning electron micrograph of 2–4-mm immature ears from Zheng58×lx9801^*hlEW2b*^ (HY) and Zheng58×lx9801 (CK), Bar = 200 μm. **B** Scanning electron micrograph of 2–4-mm immature ears from lx9801, lx9801^*hlEW2b*^ and Zheng58, Bar = 150 μm. **C**–**D** Comparison of inflorescence meristem size between lx9801, lx9801^*hlEW2b*^, Zheng58, Zheng58×lx9801^*hlEW2b*^ and Zheng58×lx9801. * and ** indicate significant differences at the 0.05 and 0.01 probability levels. More than 10 individuals were measured for each inbred line, and 30 individuals were measured for the two hybrids

To understand the developmental basis of ear width, the IM sizes of 2–4 mm immature ears were observed. The average diameter of the ear IM in the developing female inflorescence of HY was 501.21 ± 19.98 μm, significantly larger (*P*-value = 3.36E − 09) than that of CK (465.29 ± 17.04 μm) (Fig. 2A and C). However, there were no significant differences in IM length, which was determined using the final lengths of mature ears in the CK and HY hybrids. In addition, the comparison results at the inbred line level showed that there were no significant differences between lx9801 and lx9801^*hlEW2b*^ in the length and width of the IM (Fig. 2B–D), which may be caused by the similar genetic background of the near-isogenic lines (NILs, lx9801 and lx9801^*hlEW2b*^). The IM length of Zheng58 was significantly lower than that of lx9801 and lx9801^*hlEW2b*^, and the width was significantly greater than that of lx9801 and lx9801^*hlEW2b*^. We also compared IM sizes between the hybrids and their parents and found that both IM length and width were significantly higher in the hybrids (Fig. 2C–D, Supplementary Table S2). This result may be due to hybrid heterosis. Thus, the presence of the heterotic locus *hlEW2b* may significantly increase the IM widths of hybrids but not NILs. The wider IM may provide more space for ear development, leading to the significantly greater ear width and weight in HY than in the control hybrid CK.

### RNA-seq and data filtering

For comparative transcriptome analysis, 15 IM samples (three replicates each of lx9801, lx9801^*hlEW2b*^, Zheng58, CK, and HY) were used to construct cDNA libraries for RNA-seq. After reads with low-quality bases were removed, 2.05–3.41 million and 4.05–5.58 million clean reads were obtained from inbred lines and hybrids, respectively. Approximately 66.96–74.95% of the clean reads from the inbred lines and 71.56–77.96% from the hybrids were unique and could be aligned with the maize B73 reference genome (Zea_mays.B73_RefGen_v4.41; Table 2). To avoid false positive estimates for gene expression, a transcript was considered to be positively expressed only if fragments per kilobase of exon per million mapped reads (FPKM) ≥ 1. Based on this criteria, a total of 20 086, 20 816, and 20 448 genes were transcribed in Zheng58, lx9801^*hlEW2b*^, and lx9801, respectively. In addition, there were 21 703 and 21 622 genes transcribed in HY and CK hybrids, respectively. On average, 2143 and 4009 genes displayed high (FPKM ≥ 50) and medium (20 ≤ RPKM < 50) expression, respectively, and 14 783 genes exhibited low expression levels that accounted for 70.61% of the expressed genes (FPKM < 20). More genes were expressed in hybrids than in their corresponding parents in both HY and CK hybrids (Supplementary Table S3).

**Table 2:** Quality control results of RNA-seq sequencing

Sample name	Clean reads	Mapped reads	Unique reads	Overall alignment rate (%)
Zheng58×lx9801^*hlEW2b*^-1	52696924	41081662	15228972	77.96
Zheng58×lx9801^*hlEW2b*^-2	50729427	37786413	13355029	74.49
Zheng58×lx9801^*hlEW2b*^-3	46591671	33340291	22116933	71.56
Zheng58×lx9801-1	40571709	29508794	20490525	72.73
Zheng58×lx9801-2	43573400	33108175	19836565	75.98
Zheng58×lx9801-3	55896954	42325450	28002298	75.72
lx9801^*hlEW2b*^-1	26511075	18086226	13867086	68.22
lx9801^*hlEW2b*^-2	24226497	16831450	12929446	69.48
lx9801^*hlEW2b*^-3	23455267	15918412	12238590	67.87
lx9801-1	23454232	15906876	12133384	67.82
lx9801-2	23904060	16110550	12256263	67.4
lx9801-3	20571135	13774186	10530690	66.96
Zheng 58-1	25073927	18793335	15345069	74.95
Zheng 58-2	28394765	19640847	14589745	69.17
Zheng 58-3	34151444	23091536.5	16754092	67.62

### Identification of differentially expressed genes (DEGs) by RNA-seq

**Fig. 3:**
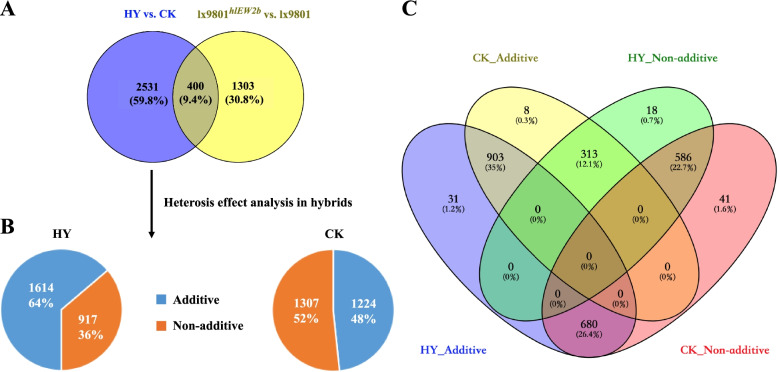
Expression patterns of differentially expressed genes (DEGs). **A** Statistical analyses of DEGs between hybrids [Zheng58 × lx9801^*hlEW2b*^ (HY), Zheng58 × lx9801 (CK)] and their corresponding inbred lines lx9801^*hlEW2b*^ and lx9801. **B** Expression pattern analysis of specific DEGs between hybrids. **C** Venn diagram showing the number of additively (HY_additive and CK_additive) and non-additively (HY_Non-additive and CK_Non-additive) expressed genes in each hybrid

To study the effect of differences in gene expression on heterosis between HY and CK, we compared their transcriptomes and identified a total of 2931 DEGs between them. Four hundred of the DEGs in the HY vs. CK comparison may result from the substitution of the *hlEW2b* fragment into the recipient parent lx9801 at the level of the inbred line, rather than from crosses of lx9801^*hlEW2b*^ and lx9801 with the test parent Zheng58. The reason for identifying DEGs between lx9801^*hlEW2b*^ and lx9801 (1703 DEGs) and excluding the 400 DEGs shared by the two pairwise comparisons of HY vs. CK and lx9801^*hlEW2b*^ vs. lx9801 from the DEG set of the HY vs. CK comparison was to eliminate interference caused by differences between the inbred lines at the hybrid level. Therefore, the following analysis focuses on the 2531 genes that were unique to the HY vs. CK comparison (Fig. 3A). Of the unique DEGs from the HY vs. CK comparison, 1261 were upregulated (HY > CK), and 1270 were downregulated (HY < CK) (Supplementary Table S4).

### Differential gene expression patterns associated with heterosis

To fully examine differences in gene expression patterns and their effects on heterosis, various pairwise comparisons were performed between hybrids and their corresponding mid-parent values (MPVs). Based on the criteria of FPKM ≥ 1 in at least one genotype and false discovery rate (FDR) < 0.05, a total of 11 808 and 9895 genes were additively and non-additively expressed in the HY hybrid. In the CK hybrid, 8909 and 12 713 genes displayed additive and non-additive expression, respectively. Because of the highly similar genetic backgrounds of the two hybrids (HY and CK share the same female paternal line Zheng58, and the male paternal lines lx9801 and lx9801^*hlEW2b*^ were near-isogenic lines), DEGs from the pairwise comparison of HY vs. CK may be responsible for the different heterotic performance between the hybrids during the female inflorescence developmental stage.

To gain overall insights into the expression patterns of unique DEGs in HY vs. CK, genes were classified as additively or non-additively expressed based on pairwise comparisons between their values in the hybrids and the corresponding MPVs. A total of 1614 DEGs (64% of 2531 unique DEGs from HY vs. CK) were expressed additively in HY, and 1224 DEGs (48% of 2531 unique DEGs from HY vs. CK) were expressed additively in CK (F_1_ vs. MPV, FDR > 0.05) (Fig. 3B). In addition, 769 upregulated (HY > CK, 47.65% of 1614 additively expressed genes in HY) and 845 downregulated (HY < CK, 52.35% of 1614 genes) additively expressed genes were identified in the HY hybrid. There were 794 upregulated (CK > HY, 64.87% of 1224 additively expressed genes in CK) and 430 downregulated (CK < HY, 35.13% of 1224 genes) additively expressed genes in the CK hybrid (Supplementary Figure S1). The prevalence of additively expressed genes implied complementary effects on gene expression in the hybrid [34]. In this study, the majority of DEGs from HY vs. CK displayed an additive expression pattern; more than two-thirds of the DEGs from HY and nearly half of the DEGs from CK were additively expressed. This result suggests that complementary effects have a fundamental role in the early formation of maize ear heterosis.

In addition, previous heterosis research at the transcriptional level has shown that genes with over-dominance and dominance exhibit a non-additive expression pattern [35, 36], implying that non-additively expressed genes could more reasonably explain classical hypotheses for heterosis, such as the over-dominance and dominance hypotheses. Here, 917 genes in HY (36% of 2531 unique DEGs from HY vs. CK) and 1307 genes in CK (52% of 2531 unique DEGs from HY vs. CK) displayed non-additive expression patterns (F_1_ vs. MPV, FDR < 0.05) (Fig. 3B). There were 484 upregulated (HY > CK, 52.78% of 917 non-additively expressed genes in HY) and 433 downregulated (HY < CK, 47.22% of 917 genes) non-additive DEGs in the HY hybrid. Likewise, there were 480 upregulated (CK > HY, 36.73% of 1307 non-additively expressed genes in CK) and 827 downregulated (CK < HY, 63.27% of 1307 genes) non-additive DEGs in the CK hybrid (Supplementary Figure S1). The non-additively expressed DEGs in the HY and CK hybrids could be further classified into over-dominant (ODO), under-dominant (UDO), lx9801^*hlEW2b*^-dominant, lx9801-dominant, Zheng58-dominant, and conserved expression classes. The detailed proportions of genes in each class in the two hybrids are listed in Table 3. In HY, 120 and 273 genes were ODO and UDO; 212 and 122 genes exhibited lx9801^*hlEW2b*^-dominance and Zheng58-dominance; and 142 genes had a conserved expression pattern (Table 3). In CK, 255 and 507 genes were ODO and UDO; 199 and 145 genes displayed lx9801-dominance and Zheng58-dominance; and 201 genes had a conserved expression pattern. Nearly half of the non-additively expressed genes were ODO and UDO in the two hybrids (HY, 42.86% ODO and UDO; CK, 58.30% ODO and UDO) (Table 3). In both the HY and CK hybrids, a greater proportion of genes showed lx9801^*hlEW2b*^-dominance and lx9801-dominance than Zheng58-dominance, implying that lx9801^*hlEW2b*^ and lx9801 alleles may strongly affect gene expression levels in the hybrids.

**Table 3:** Classification of additive and non-additive expression patterns for DEGs from HY vs. CK

DEGs from Zheng58 × lx9801^*hlEW2b*^ vs. Zheng58 × lx9801			
Zheng58 × lx9801^*hlEW2b*^	Number	Zheng58 × lx9801	Number
Additivity^a^ (F_1_=MPV)	1614(63.77%)	Additivity^a^ (F_1_=MPV)	1224(48.36%)
Non-additivity^a^ (F_1_ ≠ MPV)	917(35.23%)	Non-additivity^a^ (F_1_ ≠ MPV)	1307(51.64%)
Over-dominance^b^	120	Over-dominance^b^	255
Under-dominance^c^	273	Under-dominance^c^	507
lx9801^*hlEW2b*^-dominance^d^	212	lx9801-dominance^g^	199
Zheng58-dominance^e^	122	Zheng58-dominance^e^	145
Conserved^f^	142	Conserved^f^	201
Total	2531	Total	2531

^a^ based on fisher exact test between midparent value (MPV) and hybrid (qvalue < 0.05)

^b^ above two parsents; based on fisher exact test (FDR < 0.05) between two parents and hybrid

^c^ below two parsents; based on fisher exact test (FDR < 0.05) between two parents and hybrid

^d^ based on fisher exact test (FDR < 0.05); hybrid value must be significantly different than midparent value and not significantly different from lx9801^*hlEW2b*^ parent

^e^ based on fisher exact test (FDR < 0.05); hybrid value must be significantly different than midparent value and not significantly different from Zheng58 parent

^f^ based on fisher exact test (FDR < 0.05); hybrid value must be significantly different than midparent value and within the parental range

^g^ based on fisher exact test (FDR < 0.05); hybrid value must be significantly different than midparent value and not significantly different from lx9801 parent

### Functional characterization of additively and non-additively expressed genes

**Fig. 4:**
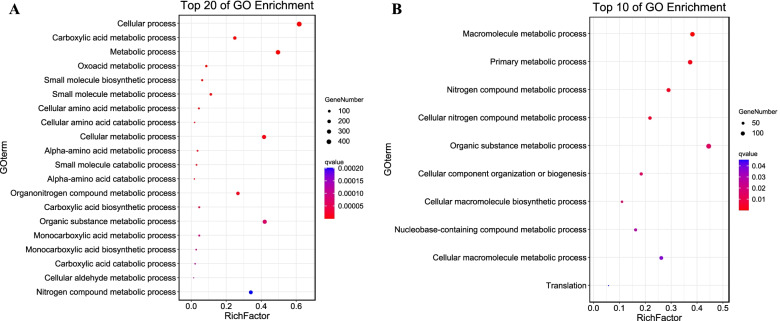
Comparison and functional enrichment of additive genes in hybrids. **A** Top 20 enriched biological process GO terms in additive genes of the HY hybrid. **B** All 10 enriched biological process GO terms in the CK hybrid. The Y-axis lists the enriched GO terms, and the X-axis represents the rich factor. The smaller the Q value, the higher the significance level and the closer to the red point. The more genes, the larger the circle

We performed a gene ontology (GO) enrichment analysis at the AgriGO website to determine the molecular and biological functions of additive and non-additive DEGs in heterotic performance and to assess their biological roles in heterosis during ear development in the hybrids. In total, 711 of 1614 and 321 of 1224 additively expressed DEGs in HY and CK, respectively (Fig. 3C), were found to be enriched (FDR < 0.05, Yekutieli FDR dependency) in terms from the three hierarchically structured GO categories: biological process (BP), molecular function (MF) and cellular component (CC). The additive DEGs specifically expressed in HY were mainly enriched in 44 BP GO terms (Supplementary Table S5), and the three most significant terms were Cellular process (GO:0009987), Carboxylic acid metabolic process (GO:0019752), and Metabolic process (GO:0008152). In the MF category, the three most significantly enriched terms were Catalytic activity (GO:0003824), ATP binding (GO:0005524), and Carbohydrate derivative binding (GO:0097367), whereas Cytoplasm (GO:0005737) and Intracellular (GO:0005622) were enriched in the CC category (Fig. 4A). In the CK hybrid, specific additive DEGs were enriched in 10 main GO terms (Fig. 4B, Supplementary Table S6). The top three most significantly enriched BP terms were Macromolecule metabolic process (GO:0043170), Primary metabolic process (GO:0044238), and Nitrogen compound metabolic process (GO:0006807). The top enriched MF terms were Binding (GO:0005488), Structural constituent of ribosome (GO:0003735), and Structural molecule activity (GO:0005198), and the top CC terms were Cytosolic ribosome (GO:0022626) and Intracellular (GO:0005622). More GO terms were enriched in the additively expressed DEGs of HY than of CK, suggesting that additively expressed genes played an important role during early development of the HY maize ear.

**Fig. 5:**
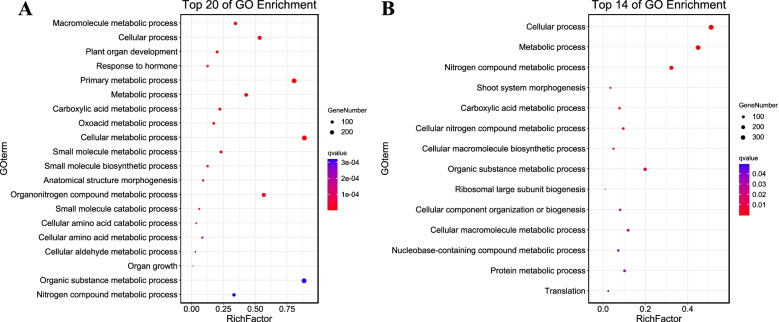
Comparison and functional enrichment of non-additive genes in hybrids. **A** Top 20 enriched biological process GO terms in non-additive genes of the HY hybrid. **B** All 14 enriched biological process GO terms in the CK hybrid. The Y-axis lists the enriched GO terms, and the X-axis represents the rich factor. The smaller the Q value, the higher the significance level and the closer to the red point. The more genes, the larger the circle

The 331 (331/917) and 721 (721/1307) non-additively expressed DEGs in HY and CK (Fig. 3C) were specifically enriched in different GO functional categories. In HY, the non-additively expressed genes were most enriched in the BP terms Macromolecule metabolic process (GO:0043170), Cellular process (GO:0009987), Plant organ development (GO:0099402), Response to hormone (GO:0009725), and Primary metabolic process (GO:0044238). They were enriched in the MF terms Binding (GO:0005488) and Structural molecule activity (GO:0005198). In the CC category, the most enriched terms were Cytosol (GO:0005829), Organelle (GO:0043226), and Cellular anatomical entity (GO:0110165) (Fig. 5A, Supplementary Table S7). Genes that were non-additively expressed in CK were enriched in BP terms such as Cellular process (GO:0009987), Metabolic process (GO:0008152), Nitrogen compound metabolic process (GO:0006807), and Shoot system morphogenesis (GO:0010016). In the MF and CC categories, the most enriched terms were Binding (GO:0005488), Catalytic activity (GO:0003824), and Small molecule binding (GO:0036094) and Cytoplasm (GO:0005737), Intracellular (GO:0005622), and Membrane-bounded organelle (GO:0043227) (Fig. 5B, Supplementary Table S8). These results may imply that multiple metabolic processes, plant organ morphogenesis, and hormone response processes, among others, play important roles in maize ear development and early heterosis formation.

### Allele-specific expression analysis

**Fig. 6:**
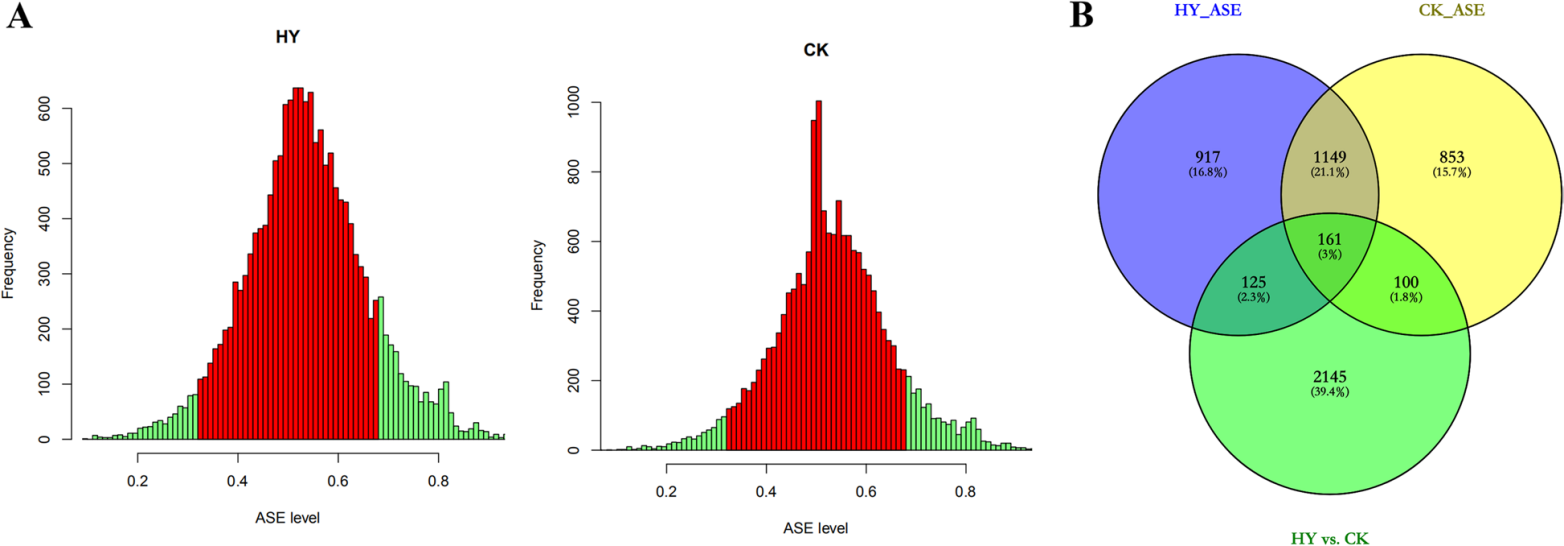
Global allele-specific expression analysis. **A**: The distribution of ASE genes in hybrids. Green and red represent genes with ASE and no ASE at the genome-wide level in Zheng58 × lx9801^*hlEW2b*^ (HY) and Zheng58 × lx9801 (CK). **B** Comparison of DEGs between hybrids that show an ASE pattern

ASE is an important source of gene expression divergence in hybrids [37]. Several studies have suggested that ASE plays a role in heterosis because genetic variation often leads to differences in gene expression, which then lead to phenotypic changes [17, 18]. Based on single nucleotide polymorphism (SNP) data and specific allele sequencing data for the parents, genes that showed ASE were identified in the hybrids. First, SAMtools was used to obtain SNP information for lx9801 and Zheng58 in the CK hybrid and for lx9801^*hlEW2b*^ and Zheng58 in the HY hybrid. A total of 362 410 and 360 791 polymorphic SNPs were identified between lx9801 and Zheng58 and between lx9801^*hlEW2b*^ and Zheng58, respectively (Fig. 6A, Table 4). Based on ASE gene identification criteria (see *Materials and methods*), 2263 genes (12.89% of 17 563 analyzed genes) and 2352 genes (14.65% of 16 059 analyzed genes) were identified as having significant allelic bias in CK and HY, respectively (Table 4).

**Table 4:** Allele-specific gene expression genes in two hybrids

Sample	SNPs for ASE analysis	Protein coding genes	Significant ASEs	ASE genes ratio (%)
Zheng58×lx9801	362410	17563	2263	12.89
Zheng58×lx9801^*hlEW2b*^	360791	16059	2352	14.65

**Fig. 7:**
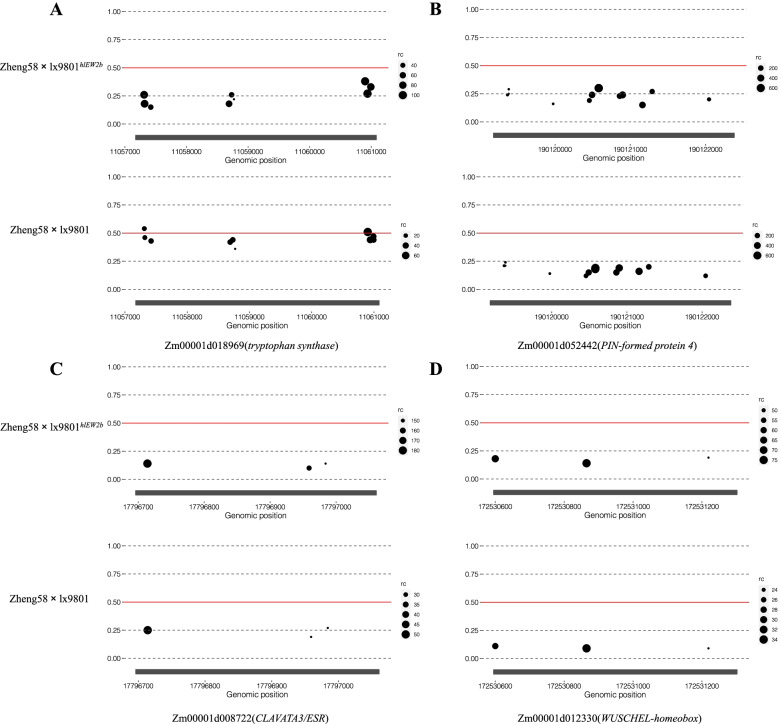
Critical DEGs with an ASE expression pattern in HY and CK hybrids that participate in regulation of ear development. Each point on the figure represents an SNP between the two parental genomes of the hybrid, and the size of the point indicates the read number at each SNP. The more reads, the larger the point. The X-axis represents the position of the SNP in the parental genomes, and the Y-axis represents the level of allele-specific expression (P). The lx9801^*hlEW2b*^ allele is set to 0 in Zheng58×lx9801^*hlEW2b*^, and the lx9801 allele is set to 0 in Zheng58×lx9801. The test parent Zheng58 is set to 1 in both hybrids

To more fully elucidate the relationship between ASE and heterosis, we identified DEGs between the hybrids that also showed ASE in HY and CK. When we compared HY ASE genes with CK ASE genes, we found that 286 genes in HY and 261 genes in CK showed both ASE and differential expression between the two hybrids. Among these DEGs, 161 showed ASE in both hybrids, 125 showed ASE only in HY, and 100 showed ASE only in CK (Fig. 6B). We performed GO analysis to determine the molecular and biological functions of these genes, but no BP or MF GO terms were significantly enriched among all three gene sets. However, some auxin synthesis and transport genes (such as *tryptophan synthase*, *PIN-formed protein*, and *brachytic2*) (Fig. 7 A–B, Supplementary Figure S2), plant growth and development genes (such as *bHLH-transcription factor* and *AP2-EREBP-transcription factor*), and inflorescence meristem development genes (such as *CLAVATA3/ESR* and *WUSCHEL-homeobox-transcription factor*) were identified among the DEGs with ASE in the HY hybrid (Fig. 7 C–D, Supplementary Table S9). Among them, *PIN-formed protein4*, *CLAVATA3/ESR*, and *WUSCHEL-homeobox-transcription factor* genes were simultaneously identified in both hybrids (Supplementary Table S10). In the set of DEGs with ASE in the CK hybrid, genes related to amino acid synthesis (such as *glutamine synthetase*) (Supplementary Figure S2), growth and developmental regulation (such as *MYB-transcription factor* and *AP2-EREBP-transcription factor*), and maintenance of phytohormone homeostasis (such as *OVATE-transcription factor*) differed in expression from the HY hybrid (Supplementary Figure S2, Supplementary Table S11). These results implied that ASE genes may participate in various biological processes, potentially leading to differences in heterotic performance.

## Discussion

### Overdominance effects may be the greatest contributors to heterosis

In recent years, many studies have shown that overdominance effects contribute to heterosis, and some genes that exhibit overdominance effects have been identified. The most typical example is the *SINGLE FLOWER TRUSS* (*SFT*) gene in tomato, which confirmed the overdominance effects of heterosis. Krieger et al. (2010) found that the *SFT* gene was heterozygous for a functional allele and that a loss-of-function allele resulted in overdominant performance for tomato yield [38]. The *FT* gene (an *SFT* homolog) controls florigen synthesis in *Arabidopsis*. When heterozygous, *FT* causes *A. thaliana* to differentiate to form more inflorescences than the parents, showing an overdominance effect for heterosis [39]. In addition, heterotic loci with overdominant effects at the single-site level have been identified using genetic mapping populations [40]. However, these putative overdominant genes or heterotic loci have only been analyzed at the single gene or locus level, without controlling for the complex genetic backgrounds of the materials themselves. Therefore, pseudo-overdominance and epistatic heterotic effects may exist at the genomic level. In this study, single-fragment substitution lines were used to eliminate the interference of the genetic background. To analyze the heterotic effects of *hlEW2b*, the ear width phenotypes of the test hybrid (Zheng58 × lx9801^*hlEW2b*^) and its parental inbred lines (Zheng58 and lx9801^*hlEW2b*^) were assessed under multiple environmental conditions, and the heterotic locus *hlEW2b* had an overdominance effect on heterosis of ear width.

### Different expression patterns play critical roles in heterosis of maize ear width

Changes in gene expression pattern may cause heterosis in hybrids [41, 42]. By analyzing transcriptome data of hybrids and parents, gene expression patterns can be divided into additive and non-additive patterns [43]. Among species with different heterotic rates (such as *Arctic char*, *Medicago*, and *Larix gmelinii*), hybrids with heterosis are more likely to show non-additive expression than hybrids without heterosis [44–46]. The increased activities of non-additively expressed genes in *Arabidopsis* promote photosynthetic capacity, cell size, and cell number, demonstrating that non-additively expressed genes may play important roles in biomass heterosis [46]. Meyer et al. [47] proposed that non-additively expressed genes can effectively improve the resource use of *Arabidopsis* seedlings and promote the enhancement of metabolic activities, which show strong heterosis in hybrids. However, in some cases, additively expressed genes are the greatest contributors to heterosis, or additive and non-additive genes may have similar effects [8]. A transcriptome study of maize ear growth and development during the spikelet and floret differentiation stages found that additive genes accounted for a greater proportion in the F_1_ hybrid and played fundamental roles in maize ear heterosis [34].

Here, a specific experimental design was used to explore the relationships between changes in gene expression pattern and heterosis. We found that 64% and 48% of the genes differentially expressed between hybrids were additively expressed in HY and CK, respectively, indicating that additively expressed genes may have had a greater effect on HY hybrid ear development. Previous studies have proposed that additive gene expression implies that the hybrid parental alleles have a strong complementary effect. This effect may neutralize the role of inferior alleles and adjust gene expression to an optimal level that promotes the hybrid advantage [23, 34]. This hypothesis is consistent with the results of the present study. In the dominant hybrid HY, more genes exhibited additive expression patterns, implying that more genes were in an advantageous expression state in HY than in the CK hybrid.

In addition, 36% and 52% of genes exhibited a non-additive expression pattern in HY and CK. Of these genes, nearly half displayed ODO and UDO in the two hybrids (Table 3). This result suggests that, among the different categories of non-additively expressed genes, ODO and UDO genes may have a particularly strong influence on heterosis. Furthermore, there were significantly more genes with lx9801^*hlEW2b*^- and lx9810-dominant patterns than with Zheng58-dominant patterns in HY and CK, respectively (Table 3), perhaps reflecting the influence of donor chromosome fragments. This result suggests that lx9801^*hlEW2b*^ and lx9801 alleles have a greater effect than Zheng58 alleles on gene expression levels in their corresponding hybrids, HY and CK. Together, these findings indicate that different hybrid gene expression patterns may make contributions to hybrid vigor.

### The role of carbohydrate and nitrogen metabolism in heterosis

Carbohydrate metabolism is an essential process that produces energy sources in the plant [48]. Some enzymes involved in carbohydrate metabolism, such as *triose phosphate isomerase* (*TPI*) and *Phosphoenolpyruvate carboxylase kinase* (*PEPK*), belong to a complex network that regulates carbon assimilation and conversion processes. *TPI* (*Zm00001d039865*, *Zm00001d008619*) and *PEPK* (*Zm00001d051156*) are involved in glycolysis, gluconeogenesis, and the Calvin cycle, thus playing a crucial role in storage reserve mobilization and carbohydrate conversions [49, 50]. *TPI* genes can promote starch synthesis, and *PEPK* can increase the efficiency of carbon fixation in crops [49, 50]. These genes were upregulated in HY (Supplementary Table S12–13, Supplementary Table S15) and may have helped to provide energy and carbon to support maize ear development in the HY hybrid.

In addition, nitrogen metabolism is closely related to protein synthesis and nitrogen assimilation process, which can provide abundant nitrogen sources for organs development [51]. In the aspect of protein synthesis, downregulation expression of *ribosome export associated 1* (*Zm00001d038475*) could inhibits cell proliferation and cell growth by affecting the biogenesis of 60 S ribosomal subunits, which can significantly reduce cell size, with cell width and cell length both decreased, and was not conducive to the growth and development of organs in maize [52]. In this study, the expression level of *Zm00001d038475* was higher in HY than CK, which may play a major positive role for HY female ear development. The *ribosome proteins* were mainly involved in the ribosomal protein synthesis and the assembly of ribosomes, which positive regulated the processes of the biosynthesis and processing of proteins [53]. The *ribosome proteins* (*Zm00001d049666*) up-regulation in HY could promote the proteins biosynthesis, thus providing the sufficient nitrogen source for female inflorescence development during early stage. In the aspect of nitrogen assimilation, *Glutathione transferase* (*Zm00001d036951*) involved in the TCA cycle, glycolysis and nitrogen assimilation, and the low expression of glutathione transferase in rice reduced primary root elongation and lateral root formation [54]. Glutamine provides the nitrogen that is required for purine and pyrimidine nucleotide synthesis, and these nucleic acid synthesis processes may be closely related to stem cell development in inflorescence meristems. *Glutamine synthetase* (*Zm00001d033747*) is a rate-limiting enzyme for the synthesis of glutamine [55]. These two genes were downregulated in CK, which perhaps lead to the nitrogen assimilation levels were lower in CK hybrid than HY hybrid (Supplementary Table S15). Above genes differed in expression between the hybrids and that they govern aspects of carbohydrate- and nitrogen-related processes might be promoted in the HY hybrid.

### Transcription factors involved in heterosis formation in maize ear development

Transcription factors play critical roles in responding to the onset of many biological processes involved in heterosis. To date, several transcription factor families were predicted to regulate downstream genes linked to hybrid vigor, including the Dof, MADS and MYB families [56, 57]. In maize a *Dof-transcription factor* 36 (*Zm00001d029512*) with highly conserved domain, have been demonstrated to positively control nutrient accumulation [58]. This gene showed higher expression in HY than in CK, implying that more nutrient may have accumulated to facilitate early maize ear development in HY. Many genes code for MADS-box family transcription factors are involved in meristem, floral organ, and vegetative development [59, 60]. Transcriptional expression level of *ZmMADS1* (*Zm00001d023955*), which plays a positive regulatory role in the development of the inflorescence for ear size [61], was up-regulated in HY for facilitating early maize ear development (Supplementary Table S12, Supplementary Table S15). In addition, available evidence suggests that in plants, MYB transcription factors can control the morphology and patterning of cells and play a key role in plant development [62]. In *Arabidopsis thaliana*, overexpression of *AtMYB24* produces short plants with poorly developed floral organs [63], suggesting that *AtMYB24* may be negatively regulate IM development. The *MYB transcription factors 53* (*Zm00001d044107*) in maize is homologous to *Arabidopsis thaliana AtMYB24*, and downregulation of *MYB transcription factors 53* in HY relative to CK may thus promote IM development (Supplementary Tables S14–15). The above analysis implies that IM heterosis performance is better in the HY than the CK hybrid, and this result may be influenced by regulation of gene expression by transcription factors.

### ASE DEGs involved in maize ear development

The specific expression of alleles and the unbalanced expression of the two parental alleles in hybrids are important causes of heterosis [37]. Allelic variation is widespread in genomes and is a prerequisite for studying ASE. The specific combinations of parental allelic variations in hybrids may cause changes in hybrid gene expression patterns that contribute to heterosis formation [23]. A competitive transcriptional relationship between the two alleles may be the cause of differential gene expression in hybrids. By comparing genes that were differentially expressed between hybrids, we identified 125 DEGs that showed ASE only in HY, 100 genes that showed ASE only in CK, and 161 DEGs that showed ASE in both hybrids. Gene functional annotation revealed that DEGs that displayed ASE functioned in aspects of plant hormone metabolism and signal transduction and in the regulation of IM development.

The ASE genes *AP2-EREBP-transcription factor 109* and *PIN-formed protein4*, which function in plant hormone synthesis, transport, and signal transduction processes, were identified in both hybrids (Supplementary Table S10). *AP2-EREBP-transcription factor 109* acts downstream of auxin signaling induced by low concentrations of auxin [64]. The downregulated expression of this gene in HY implies that there may be higher auxin levels in the IM of the HY hybrid than of the CK hybrid (Supplementary Table S10). Transcript expression levels of *PIN-formed protein4* (*Zm00001d052442*), a transmembrane protein that accelerates auxin transport and promotes cell elongation [65], were upregulated in HY (Fig. 7, Supplementary Table S10). A gene encoding tryptophan synthase (*Zm00001d018969*), which participates in auxin synthesis, differed in expression between the two hybrids and showed ASE only in HY (Fig. 7, Supplementary Figure S3). Tryptophan synthase participates directly in the tryptophan-dependent auxin biosynthetic pathway; by regulating the synthesis of the auxin precursor tryptophan, it is an important determinant of auxin biosynthesis [66]. Its low expression level in CK may have hindered auxin biosynthesis and restricted the development of female IMs in maize.

*WUSCHEL-homeobox-transcription factor* (*Zm00001d012330*) and *CLAVATA3/ESR-related26* (*Zm00001d008722*), which function in the regulation of IM development, showed ASE in both hybrids (Fig. 7). The former gene encodes a key transcription factor that can move via plasmodesmata into the apical domain (called the central zone) to promote the proliferation of stem cells, thereby regulating development of the shoot meristem [26]. The latter gene is negatively regulated by *WUSCHEL-homeobox-transcription factor*; it plays an important role in regulating the architecture of the female maize IM and interacts with auxin signaling [67]. The upregulation of *WUSCHEL-homeobox-transcription factor* (*Zm00001d012330*) in HY may have led to downregulation of *CLAVATA3/ESR-related26* (*Zm00001d008722*) (Supplementary Figure S3); this combined gene expression pattern may have promoted heterosis in HY during the female spike developmental stage. In addition, *OVATE family protein* (*Zm00001d022446*) is a plant-specific transcription factor that positively regulates the longitudinal diameter of wild tomato fruit [68]. Transcript expression levels of *Zm00001d022446* were upregulated and showed ASE in HY (Supplementary Figure S2, Supplementary Table S9), suggesting that this gene may encode a transcription factor that increases the diameter of the female IM in maize. ASE is an important source of gene expression differences in hybrids [69]; it may be responsible for the differential expression of the above genes between the hybrids, and these genes participate in diverse biological processes (such as plant hormone metabolism and meristem development) that influence heterosis formation during IM development.

### Auxin may participate in heterosis formation during maize ear development

**Fig. 8:**
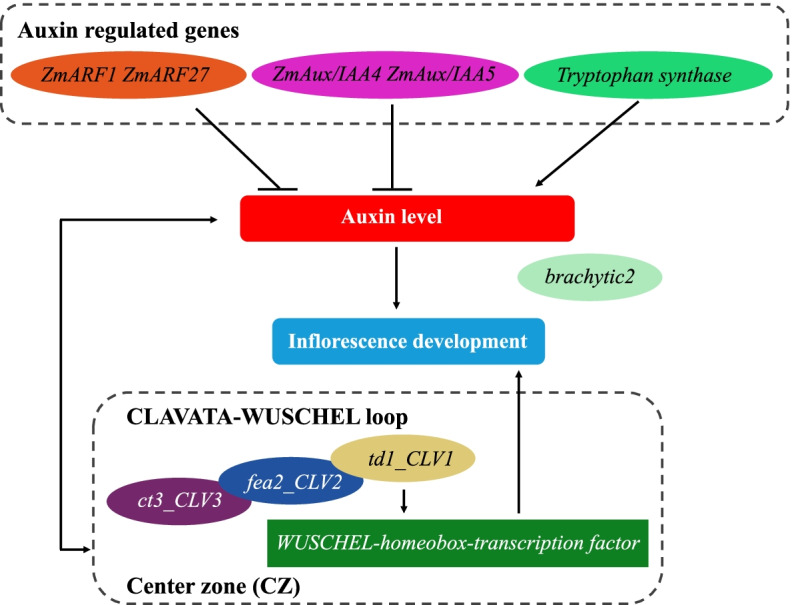
Predicting the regulatory process of heterosis formation during the inflorescence meristem period. Auxin may contribute to hybrid heterosis during maize ear development. Additive, non-additive, and allele-specific expression patterns may fine-tune the expression of crucial genes that control auxin metabolism and IM development to an optimal level, and this may be responsible for maize ear heterosis formation in hybrids

Morphological analyses have shown that the most obvious feature of the transformation from vegetative to reproductive growth is a rapid increase in the size of the IM. The cells located in the central zone of the IM have typical stem cell characteristics, and they can initiate and determine the developmental processes of maize ears [70]. The *CLV–WUS* negative feedback loop may affect the development of the IM by regulating the relationship between stem cell proliferation and tissue and organ differentiation. In this pathway, the *WUS* gene, located in the organizing center of the meristem, activates the expression of the signal molecule *CLV3*, which is sensed by *CLV1-CLV2*, and they form a complex. This complex positively regulates the expression of the *WUS* gene and promotes stem cell proliferation. However, *CLV1*, *CLV2*, and *CLV3* in the noncomplexed state inhibit *WUS* gene expression and form a dynamically balanced negative feedback loop that affects IM differentiation processes [26]. The genes involved in the *CLV–WUS* negative feedback loop have also been cloned in maize; they include *thick tassel dwarf1* and *fasciated ear2*, which are homologs of *CLV1* and *CLV2*, respectively. The homolog of *CLV3* in maize is *CLAVATA3/ESR*. The expression of *WUS* may be induced by auxin, and *WUS* gene expression is positively correlated with the auxin gradient, implying that auxin regulation is critical for IM development [71]. In the present study, several critical genes involved in inflorescence development and auxin metabolism were identified. The *thick tassel dwarf1* and *fasciated ear2* genes exhibited non-additive expression in both CK and HY (Supplementary Figure S3, Supplementary Tables S14–15) and were separately enriched in different GO terms in the two hybrids. The *CLAVATA3/ESR* gene not only showed significant differential expression between the hybrids but also showed ASE in both hybrids (Fig. 7, Supplementary Figure S3). The specific expression patterns of these genes may be responsible for the different gene expression levels between the hybrids. Our results suggest that in the IM, upregulated *CLV1* [*tassel dwarf1* (*Zm00001d014793*), HY vs. CK, FDR = 1.66E − 02, log_2_(HY/CK) = 0.57] and *CLV2* [*fasciated ear2* (*Zm00001d051012*), HY vs. CK, FDR = 2.20E − 02, log_2_(HY/CK) = 0.54] combine with *CLV3* [*CLAVATA3/ESR* (*Zm00001d008722*), HY vs. CK, FDR = 1.27E − 02, log_2_(HY/CK) = − 1.16] to form a complex, which enhances *WUS* gene expression [*WUS* (*Zm00001d012330*), HY vs. CK, FDR = 3.34E − 02, log_2_(HY/CK) = 1.16] to maintain the size of the stem cell population and positively regulate IM development (Fig. 8, Supplementary Figure S3). Rodriguez et al. (2016) and Perales et al. (2016) proposed a model in which *WUS* dimers negatively regulate *CLV3* expression in the organizing center and *WUS* monomers positively regulate *CLV3* expression in the central zone [72, 73]. This may explain why *CLV3* shows significant downregulation in the dominant hybrid HY (Supplementary Figure S3).

Local auxin gradients may play roles in the establishment of organs in the IM [74]. Several auxin synthesis and transport genes, such as *auxin response factor* (*ARF*) and *Aux/IAA* transcription factor genes, as well as functional genes encoding *brachytic2* and *tryptophan synthase* with non-additive expression patterns were all specifically enriched in the GO term Response to hormone in the HY hybrid (GO: 0009725, Response to hormone) (Supplementary Figure S3, Supplementary Table S14), and their expression differed significantly between HY and CK. The expression of *brachytic2*, which is involved in polar auxin transport [75], was significantly higher in HY than in CK. The tryptophan pathway is critical for auxin synthesis, and *tryptophan synthase* is the rate-limiting enzyme in this pathway [66]; its gene was significantly upregulated in HY compared with CK. In terms of transcription factors, ARF proteins form heterodimers with Aux/IAA proteins at high auxin concentrations, repressing the transcriptional activities of *ARF*s and *Aux/IAA* (Supplementary Figure S3) [76]. Therefore, we inferred that lower transcriptional activity of *ARF*s and *Aux/IAA* in HY may have promoted the accumulation of auxin in the IM. These results imply that auxin accumulation in the IM may be higher in HY than in CK, thus promoting greater heterosis in HY (Fig. 8).

## Conclusions

We used RNA-seq to systematically investigate the global transcriptomes of near-isogenic hybrids and their corresponding parents during the maize ear IM developmental stage. Our results identified DEGs that are involved in various carbohydrate metabolic processes, protein synthesis, and nitrogen assimilation, which may lead to phenotypic differences in the two near-isogenic hybrids. Differential expression of these genes may be the reason for different heterosis performances of HY and CK hybrids during female inflorescence development. In addition, we found evidence that auxin affects the development of the IM in maize. Our results implied that different gene expression patterns of the DEGs may fine-tune the expression of genes involved in crucial biological processes that control female inflorescence development to an optimal level, and this may be responsible for heterosis in maize ear formation in hybrids.

## Materials and methods

### Plant materials

The single-segment substitution line lx9801^*hlEW2b*^, which contains the heterotic locus *hlEW2b* associated with ear width, was developed in a previous study [33]; lx9810 and Zheng58 are leading self-lines used for maize breeding in the Yellow and Huai Valley of China. The lx9801^*hlEW2b*^ line, the receptor parent lx9801, the test parent Zheng58, and their corresponding hybrids were grown in experimental fields in Zhengzhou (Henan, China; E113°65ʹ, N34°76ʹ) in the summer of 2016. Based on the leaf-age index, 2–4-mm immature maize ear samples were collected from each plant (only the first ear was collected per plant). A microscope was used to observe and determine the developmental period. Immature maize ears were manually collected at the IM differentiation stage. There were three biological replicates for each genotype, and each biological replicate contained at least 30 immature maize ears. All samples were immediately frozen in liquid nitrogen and stored at − 80 °C.

### RNA extraction and transcriptome sequencing

Total RNA was extracted from each sample with the TRIzol reagent (Invitrogen, Carlsbad, CA, USA), and DNaseI was used to degrade the remaining DNA after extraction. The quality of the total RNA was determined using a Bioanalyzer 2100 system (Agilent Technologies, CA, USA). An Illumina TruSeq RNA sample preparation kit (Illumina, San Diego, CA, USA) was used to construct a library from each RNA sample. The prepared libraries were sequenced on the Illumina HiSeq 2000 platform. Internal Perl scripts were used to remove linker sequences and low-quality sequences (with lengths < 120 bp) [77]. After the raw data were subjected to quality control measures, the paired-end clean reads were aligned to the maize B73 reference genome (Zea_mays.AGPv4.37) using Bowtie2 software (http://bowtie-bio.sourceforge.net/bowtie2/manual.shtml) with the parameter ‘-bowtie2-N 1’, which allows for only one base mismatch during the alignment process. Cufflinks (http://cole-trapnell-lab.github.io/cufflinks/) was used to assemble transcripts, and FPKM values were used to estimate gene expression levels.

### DEG identification and gene expression pattern analysis

DEGs between samples were identified using the default parameters of Cuffdiff (a Cufflinks software component). This software controlled the FDR of the *P*-value using the Benjamini and Hochberg method and set the differential expression threshold to FDR < 0.05. To gain overall insights into gene expression patterns in the F_1_ hybrids, they were compared with MPVs. The threshold value was set to 0.05. Thus, FDR < 0.05 indicated a non-additive expression pattern, and FDR > 0.05 indicated an additive expression pattern. The GO (http://www.geneontology.org/) database was used for functional analysis of the genes. Statistically enriched GO terms in gene sets were identified using the single enrichment analysis tool at the AgriGO2 website (http://systemsbiology.cau.edu.cn/agriGOv2/); the confidence criterion was FDR < 0.05.

### ASE gene identification

Transcriptome data were used to identify SNPs at the mRNA level between the parents. SAMtools (http://www.htslib.org/doc/1.2/samtools.html) was used to identify hybrid parental SNP information. The ‘-sort’ parameter in SAMtools was used to sort the output results of Bowtie2 and convert them into a ‘bam’ file. Parental ‘bam’ files were imported into SAMtools, and the ‘mpileup’ and ‘bcftools’ parameters were used to identify SNP information between the parents [78]. The “-t DP” parameter was used to output the count results for the reference and nonreference genotypes of the SNP loci. Each parent was independently compared with the reference genotype to obtain the SNP count results between the parents.

RNAmodel is an R program (http://cran.r-project.org/) that is used to identify ASE genes at the genome-wide level and to infer the results of allelic genotype counts for specific loci [79]. For the hybrids, the allele counts of each parent gene were imported into RNAmodel, and the alleles of both parents were quantitatively estimated to determine the expression levels of different alleles. The allele-specific expression level (P) from a paternal or maternal allele was calculated based on the number of reads for the given allele divided by the total number of reads for the SNP. When P = 0, only the allele of lx9801 (Zheng58 × lx9801, CK) or lx9801^*hlEW2b*^ (Zheng58 × lx9801^*hlEW2b*^, HY) was expressed in the hybrid. When P = 1, only the allele of Zheng58 was expressed. When P = 0.5, no allele-specific expression occurred. Reliable ASE genes in the hybrids were identified according to the following criteria: (1) all reads uniquely match on the lx9801^*hlEW2b*^, lx9801, and Zheng58 genomes; (2) all reads from one parent produce a consensus base at the SNP position, different from the other parent; (3) comparisons for two alleles at each SNP position were calculated using the Benjamini–Hochberg FDR criterion, and FDR < 0.05 was used as the significance threshold; (4) there are at least two SNPs in a single gene; (5) the allele-specific expression level (P) is greater than 0.6 or less than 0.4 [37, 79, 80]. Genes that met the above criteria were identified as ASE genes.

## Supplementary Information

**Additional file 1: Supplementary Table S1.** Maize ear traits analysis of lx9801 and lx9801^*hlEW2b*^ in two environments. **Supplementary Table S2.** Comparison of inflorescence meristems size between lx9801, lx9801^*hlEW2b*^, Zheng58, HY and CK with immature ears. **Supplementary Table S3.** Distribution of gene expression levels in each sample. **Supplementary Table S4. **Differentially expressed genes between HY and CK hybrids. **Supplementary Table S5.** GO enriched terms of additive expressed genes in the Zheng58 × lx9801^*hlEW2b*^hybrid. **Supplementary Table S6.** GO enriched terms of additive expressed genes in the Zheng58 × lx9801 hybrid. **Supplementary Table S7.** GO enriched terms of non-additive expressed genes in the Zheng58 × lx9801^*hlEW2b*^ hybrid. **Supplementary Table S8. **GO enriched terms of non-additive expressed genes in the Zheng58 × lx9801 hybrid. **Supplementary Table S9.** Maker DEGs with ASE expression pattern in HY hybrid involved in regulation of ear development. **Supplementary Table S10.** Maker DEGs with ASE expression pattern in both HY and CK hybrids involved in regulation of ear development. **Supplementary Table S11.** Maker DEGs with ASE expression pattern in CK hybrid involved in regulation of ear development. **Supplementary Table S12.** Maker DEGs with additive expression pattern in HY hybrid involved in regulation of ear development. **Supplementary Table S13.** Maker DEGs with additive expression pattern in CK hybrid involved in regulation of ear development. **Supplementary Table S14.** Maker DEGs with non-additive expression pattern in HY hybrid involved in regulation of ear development. **Supplementary Table S15.** Maker DEGs with non-additive expression pattern in CK hybrid involved in regulation of ear development.

**Additional file 2: Supplementary Figure S1.** Different expression patterns DEGs up- and down-regulation relationship between two near-isogenic hybrids.

**Additional file 3: Supplementary Figure S2.** Critical DEGs with an ASE expression pattern in HY and CK hybrids that participate in regulation of ear development.

**Additional file 4: Supplementary Figure S3.** Expression analysis of marker genes in the hybrids.

## Data Availability

The datasets necessary for supporting the results of this article are included in this manuscript and its additional files. The RNA-seq datasets were deposited at the NCBI Sequence Read Archive under the accession PRJNA772971 (https://www.ncbi.nlm.nih.gov/bioproject/PRJNA772971/).

## Abbreviations

ASE: Allele-specific expression
BP: Biological process
CC: Cellular component
CK: Zheng58 × lx9801
DEGs: Differentially expressed genes
FDR: False discovery rate
FPKM: Fragments per kilobase of exon per million mapped reads
GO: Gene ontology
HY: Zheng58 × lx9801^*hlEW2b*^
IM: Inflorescence meristem
MF: Molecular function
MPV: mid-parent value
P: Allele-specific expression level
PEPK: Phosphoenolpyruvate carboxylase kinase
RNA-seq: RNA sequencing technology
SAM: Shoot apical meristem
SCF: Skp1/Cullin/F-box components
SNP: Single-nucleotide polymorphism
TPI: triose phosphate isomerase
UPS: Ubiquitin-proteasome system
