# Supplementary figures and images for: Comparative transcriptomic analysis of maize ear heterosis during the inflorescence meristem differentiation stage
Source: BMC Plant Biol. 2022 Jul 18;22:348. doi: 10.1186/s12870-022-03695-6 (PMC9290290; doi:10.1186/s12870-022-03695-6)

**Zheng58  $\times$  lx9801<sup>hIEW2b</sup>**

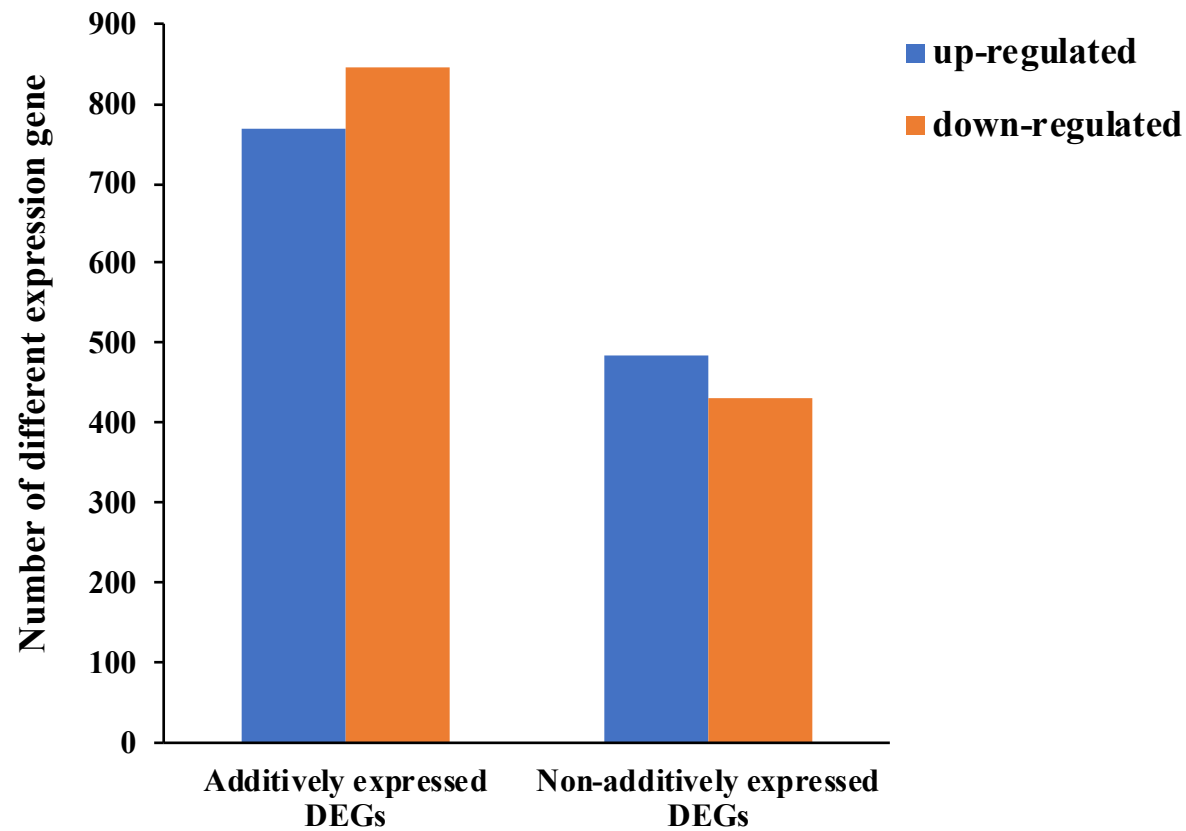

**Zheng58  $\times$  lx9801**

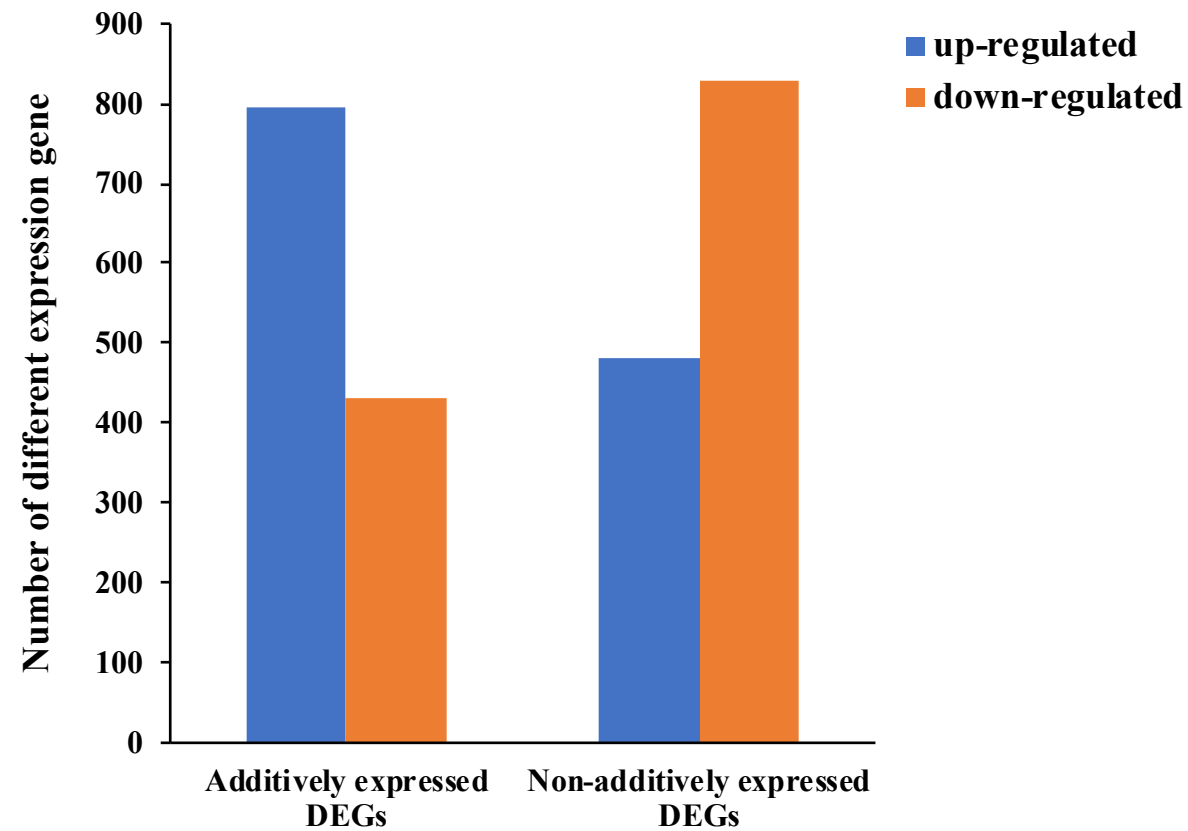

Supplement: Supplementary file 2 — Additional file 2: Supplementary Figure S1. Different expression patterns DEGs up- and down-regulation relationship between two near-isogenic hybrids. [file 12870_2022_3695_MOESM2_ESM.pdf]

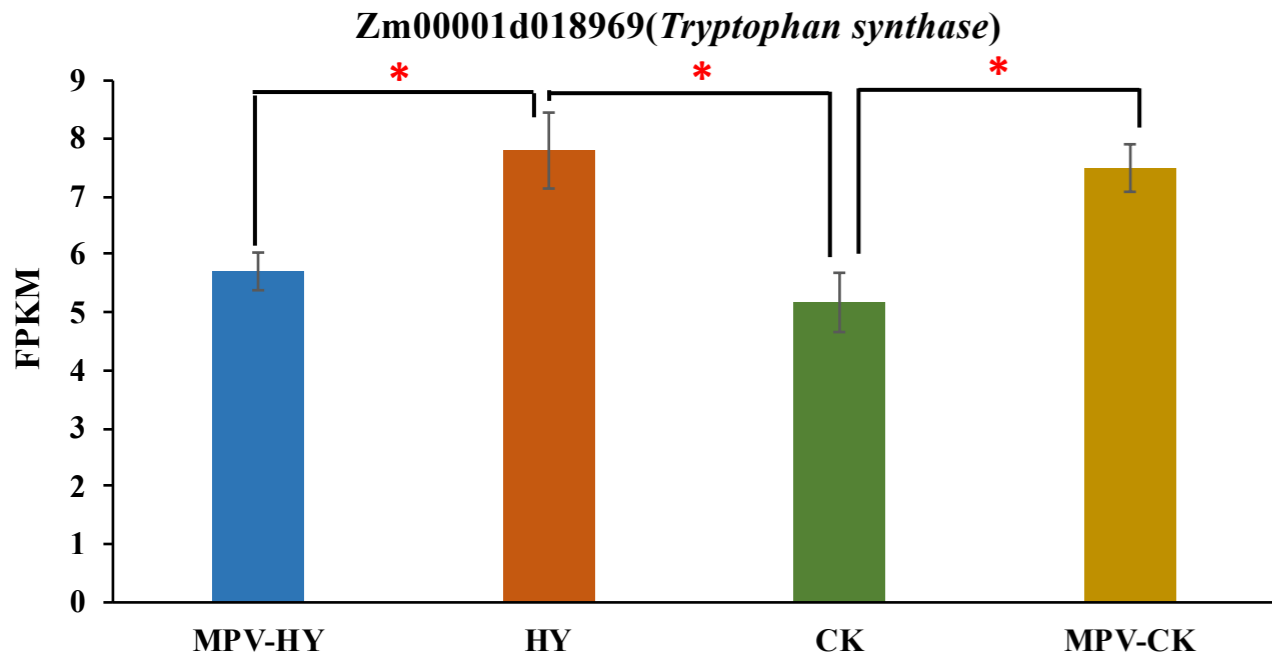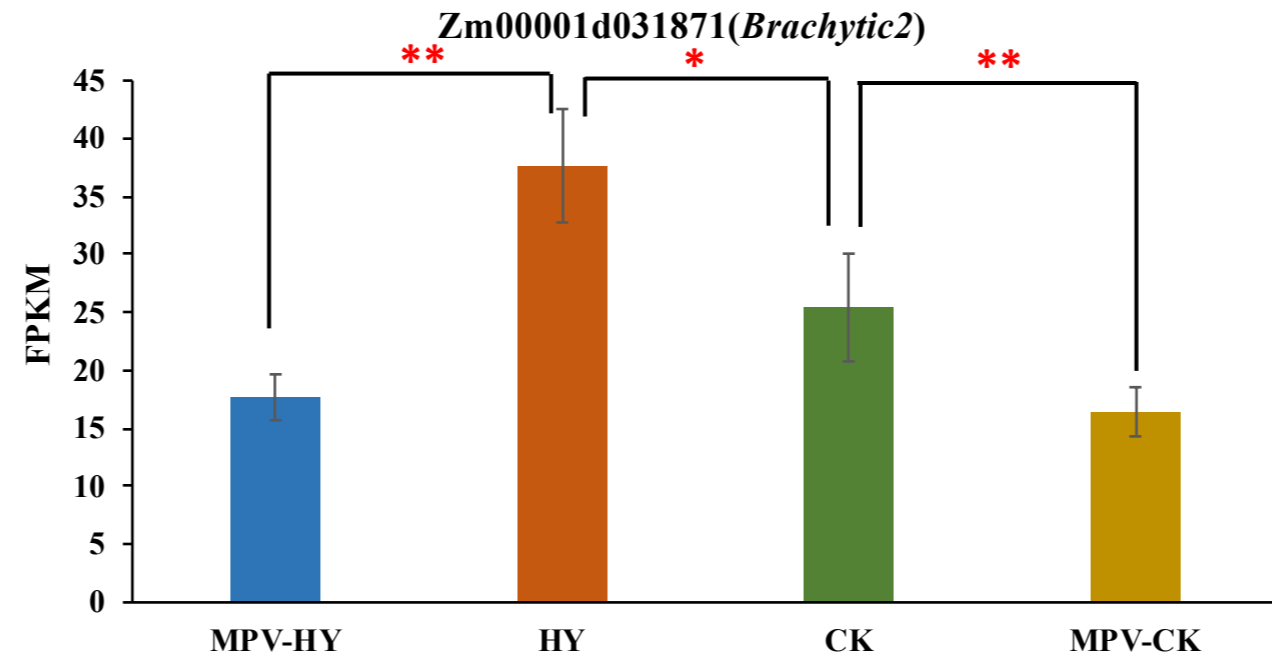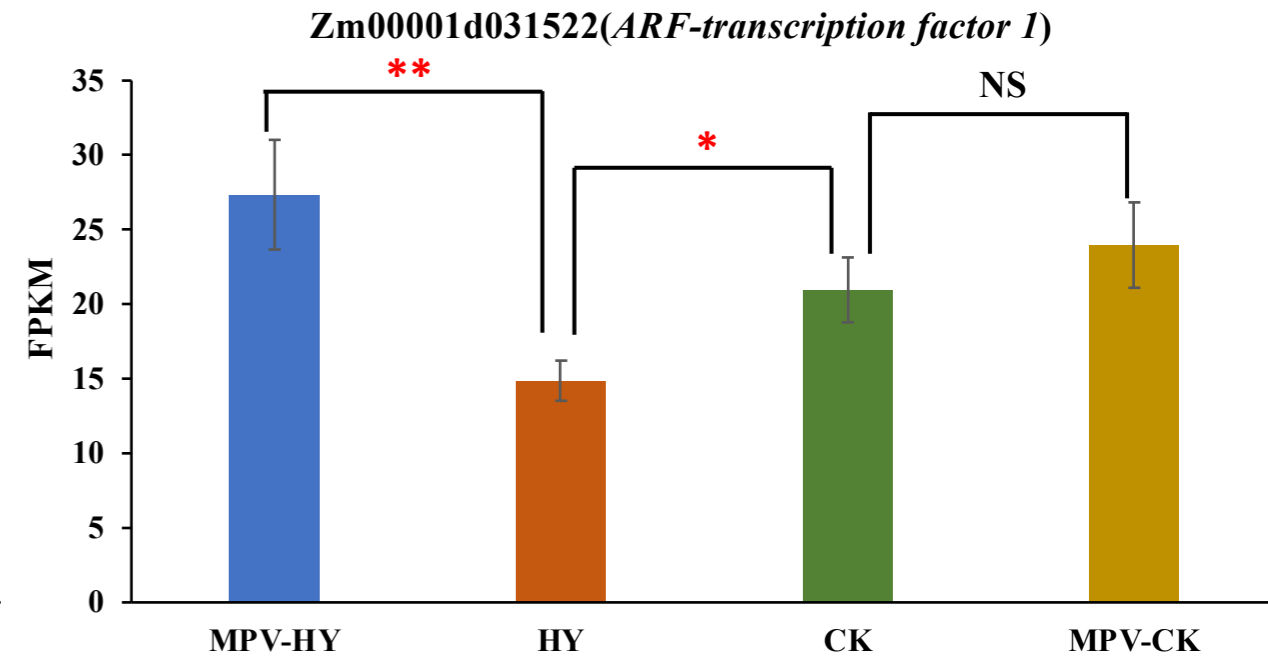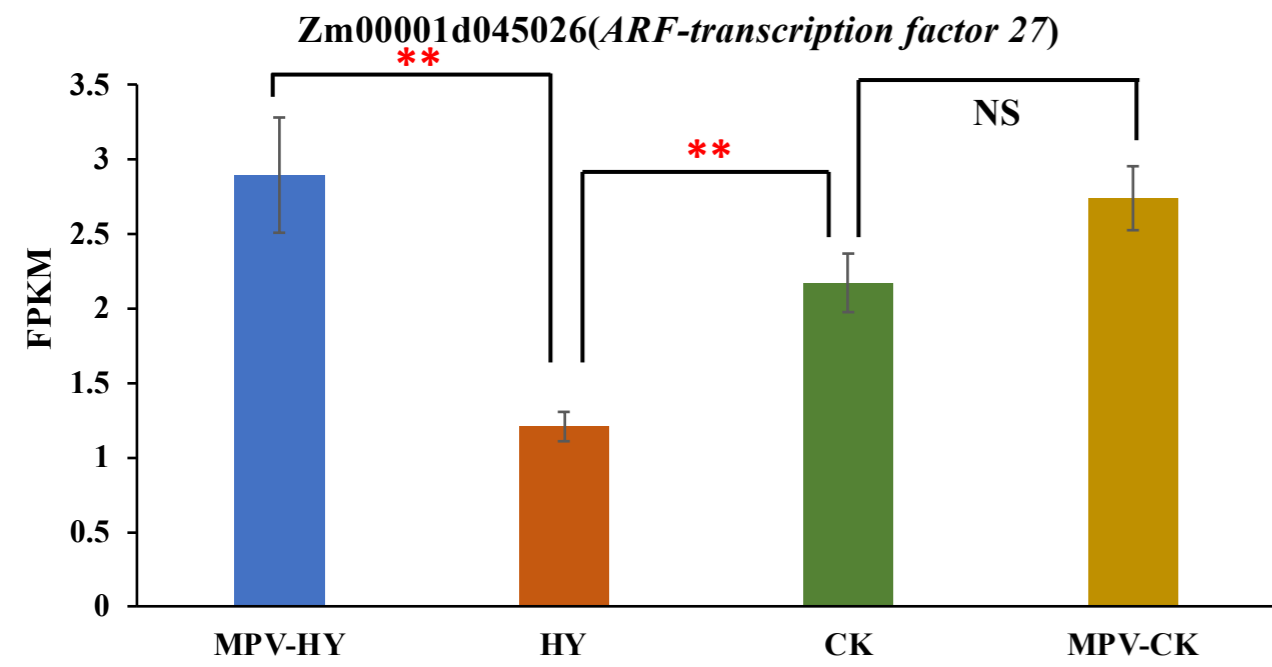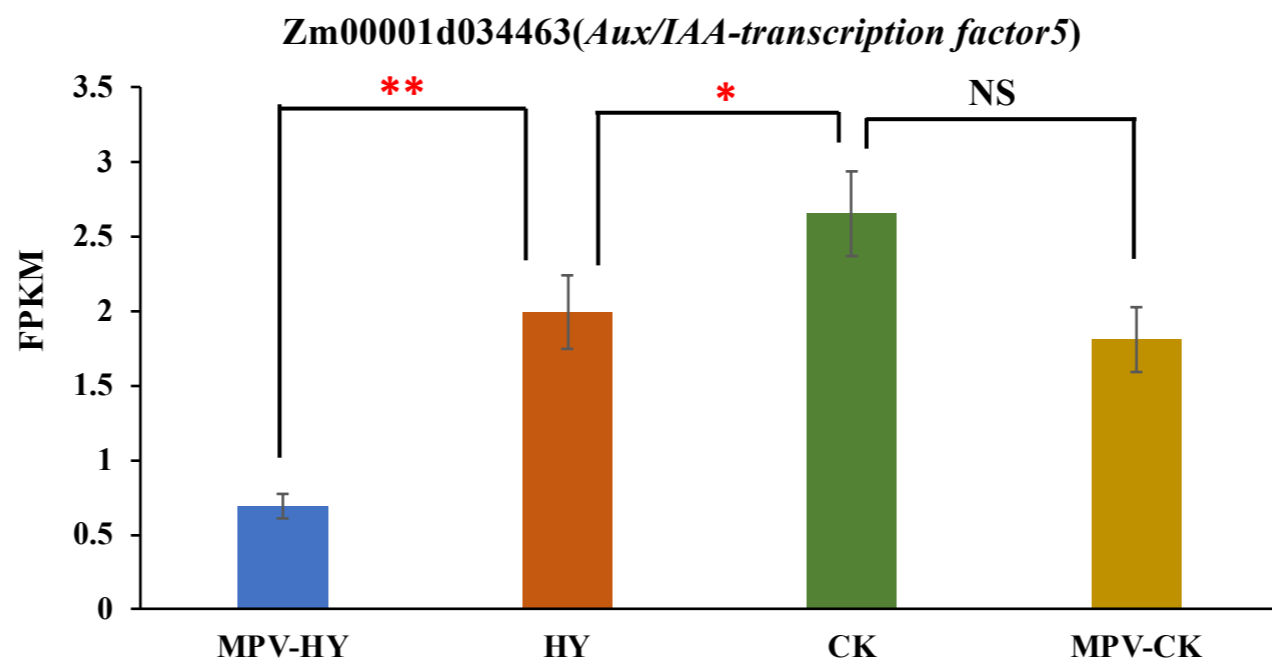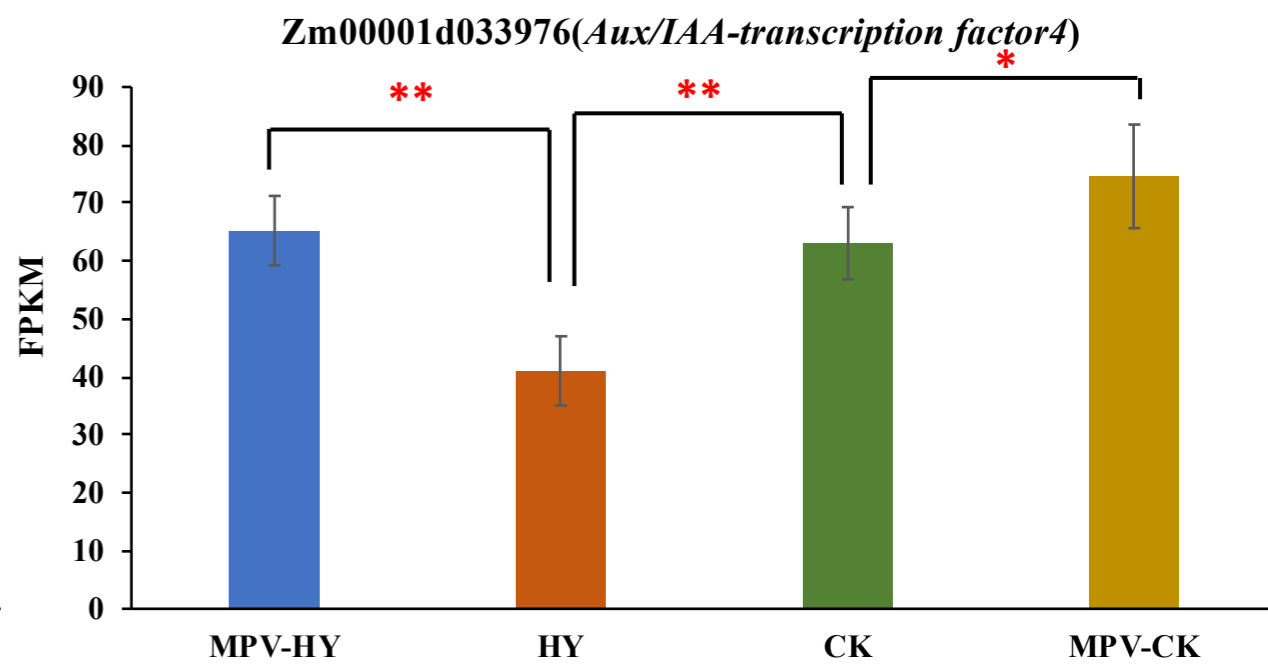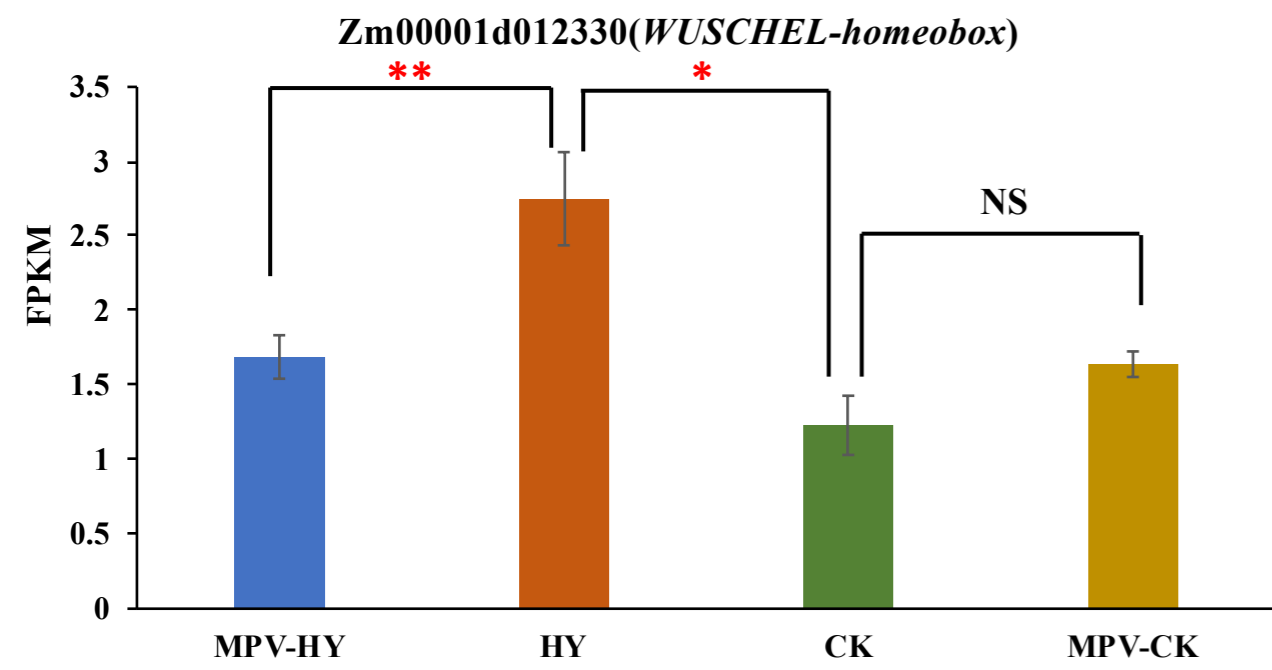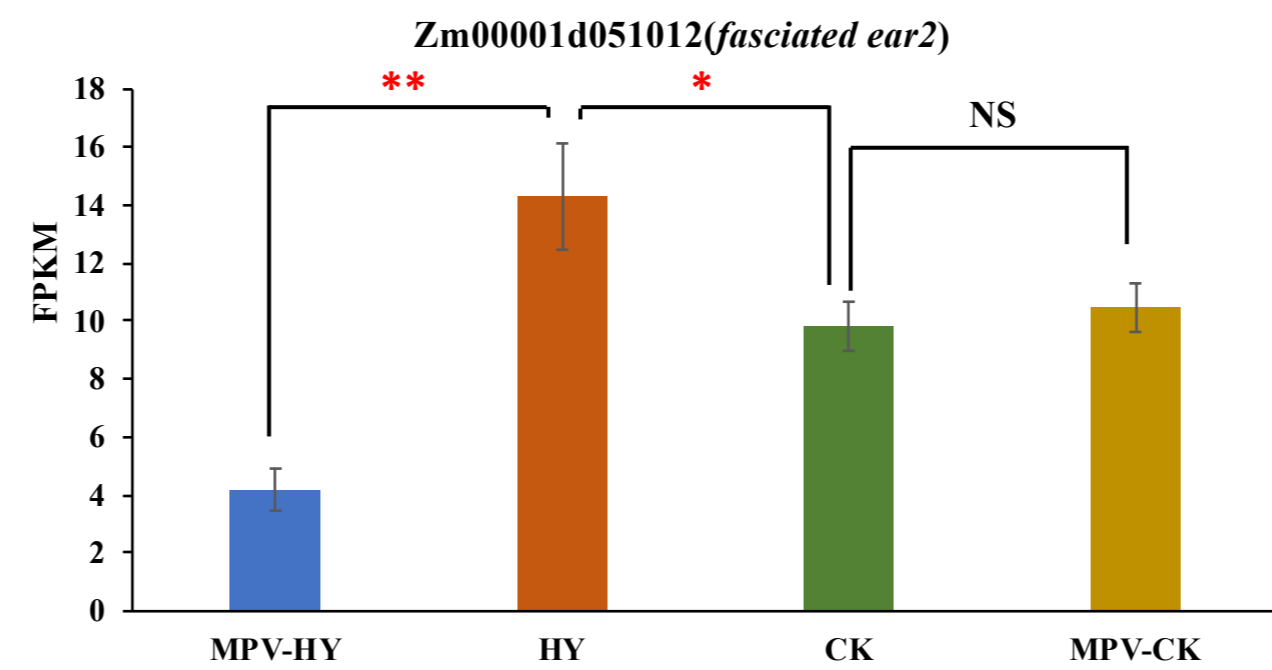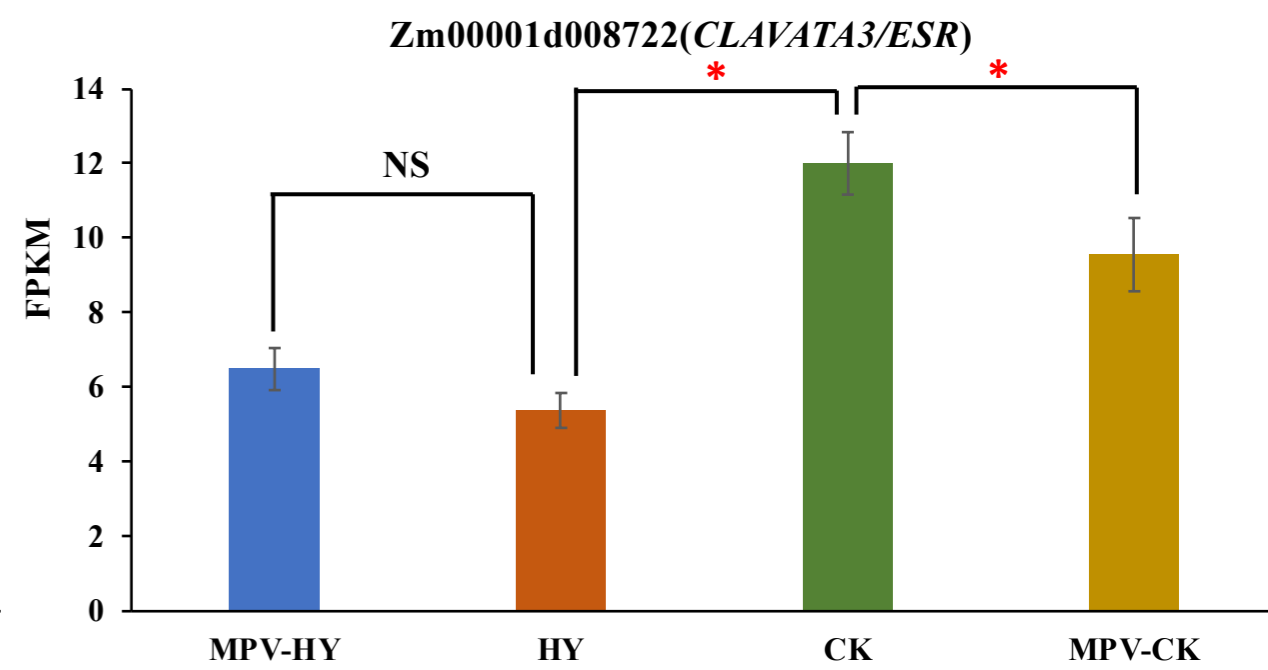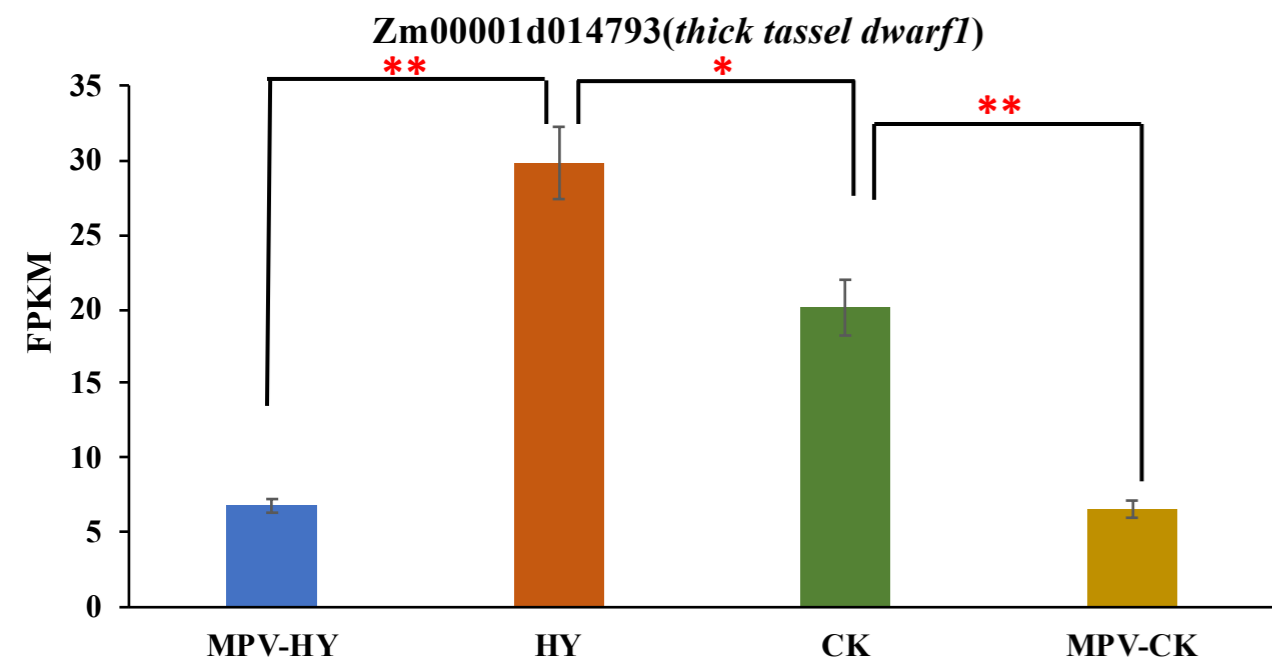

Supplement: Supplementary file 4 — Additional file 4: Supplementary Figure S3. Expression analysis of marker genes in the hybrids. [file 12870_2022_3695_MOESM4_ESM.pdf]
