# Supplementary material for: Comparative transcriptomic analysis of maize ear heterosis during the inflorescence meristem differentiation stage
Source: BMC Plant Biol. 2022 Jul 18;22:348. doi: 10.1186/s12870-022-03695-6 (PMC9290290; doi:10.1186/s12870-022-03695-6)
Supplement: Supplementary file 3 — Additional file 3: Supplementary Figure S2. Critical DEGs with an ASE expression pattern in HY and CK hybrids that participate in regulation of ear development. [file 12870_2022_3695_MOESM3_ESM.pdf]

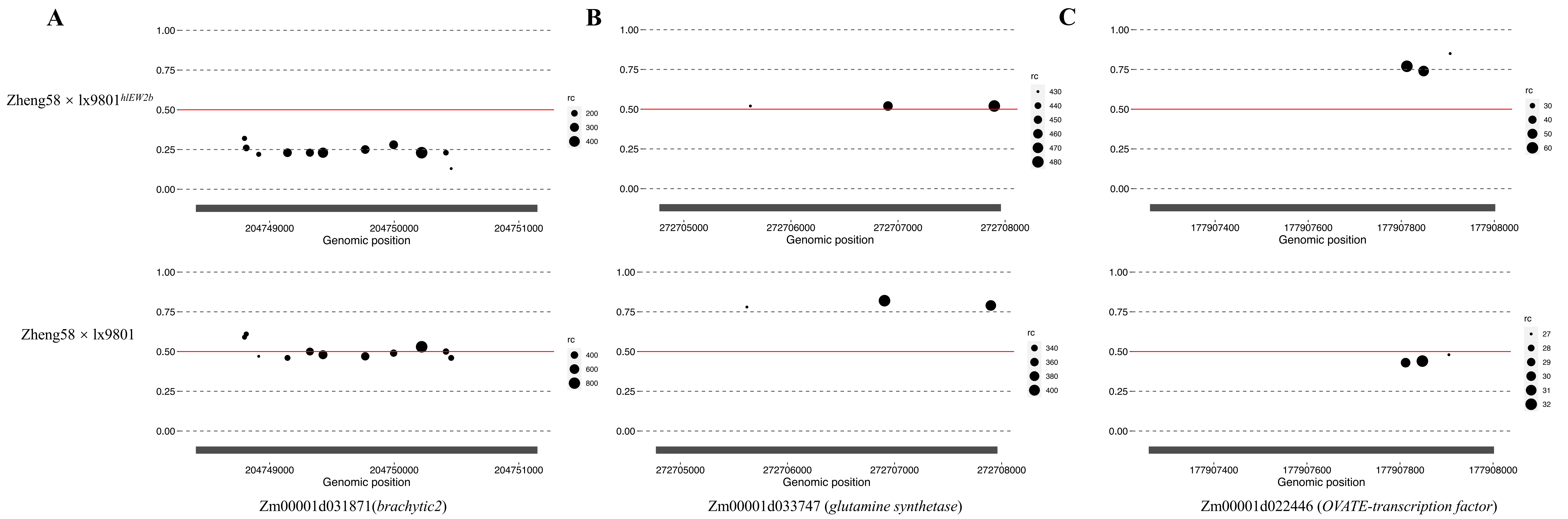
A
B
C
Zheng58 × lx9801hlEW2b
Zheng58 × lx9801
1.00
0.75
0.50
0.25
0.00
rc
Genomic position
204749000
204750000
204751000
272705000
272706000
272707000
272708000
177907400
177907600
177907800
177908000
Zm00001d031871(brachytic2)
Zm00001d033747 (glutamine synthetase)
Zm00001d022446 (OVATE-transcription factor)
